# When predictions are used to allocate scarce health care resources: three considerations for models in the era of Covid-19

**DOI:** 10.1186/s41512-020-00079-y

**Published:** 2020-05-20

**Authors:** David M. Kent, Jessica K. Paulus, Richard R. Sharp, Negin Hajizadeh

**Affiliations:** 1 Predictive Analytics and Comparative Effectiveness (PACE) Center, Tufts Medical Center, Boston, MA USA; 2 Biomedical Ethics Program, Mayo Clinic, Rochester, MN USA; 3 Feinstein Institutes for Medical Research, Northwell Health, New York City, NY USA

**Keywords:** Clinical prediction models, Covid-19, Healthcare rationing, Algorithmic fairness

## Abstract

**Background:**

The need for life-saving interventions such as mechanical ventilation may threaten to outstrip resources during the Covid-19 pandemic. Allocation of these resources to those most likely to benefit can be supported by clinical prediction models. The ethical and practical considerations relevant to predictions supporting decisions about microallocation are distinct from those that inform shared decision-making in ways important for model design.

**Main body:**

We review three issues of importance for microallocation: (1) Prediction of benefit (or of medical futility) may be technically very challenging; (2) When resources are scarce, calibration is less important for microallocation than is ranking to prioritize patients, since capacity determines thresholds for resource utilization; (3) The concept of group fairness, which is not germane in shared decision-making, is of central importance in microallocation. Therefore, model transparency is important.

**Conclusion:**

Prediction supporting allocation of life-saving interventions should be explicit, data-driven, frequently updated and open to public scrutiny. This implies a preference for simple, easily understood and easily applied prognostic models.

## Background

During the Covid-19 pandemic, the need for life-saving interventions, particularly mechanical ventilation and extracorporeal membrane oxygenation (ECMO), threatens to outstrip available resources in some settings. A cogent ‘multiprincipled’ approach to rationing health care resources during the current crisis has been distilled from decades of debate on the difficult subject of microallocation of scarce health care resources [1]. First among these principles is prioritizing patients to save ‘the most lives and… [maximize] post-treatment length of life.’

Yet how is this to be done? Because prognostication by physician clinical judgment is vulnerable to myriad cognitive biases [2] and prone to error [3, 4], and because the extreme psychological burdens of this approach to allocation of life-saving resources should be avoided, there is an important potential role for prognostic models. Prognostic models can offer actuarial objectivity for critical decision-making, ameliorating the influence of human biases. Many Covid-specific models have already been developed, although an early review indicates they have a high risk of bias [5]. In this viewpoint, we highlight methodological aspects of prediction that are of special relevance for allocating life-preserving interventions and have received little attention in more general methodological guidance for clinical prediction modeling [6, 7]. We believe the considerations we note may be underappreciated, as they do not apply (or may be less salient) in more typical clinical contexts that employ prediction for shared decision-making with patients.

## Main text

### Prediction of potential benefit (or of medical futility) is technically challenging

What predictions could be useful in the setting of rationing a potentially life-saving but scarce resource? Under a utilitarian framework, the prediction of interest is the probability of *benefit* (i.e. probable outcomes *with* versus *without* ventilator support). However, forecasting causal counterfactuals is almost impossible to do reliably from non-randomized data [8]. Instead, prognosis is used as an imperfect surrogate to predict the potential for benefit.

While more generally it is assumed that patients at highest risk derive the most benefit from medical interventions, amongst the critically ill, this assumption is turned on its head: medical futility (i.e. dismal prognosis *despite* maximal therapy) is typically thought to be the most useful prediction for withholding of scarce critical care resources. This approach relies on an implicit assumption of uniformly poor outcomes in the absence of intervention.

However, medical futility—like benefit—is also technically very difficult to predict with sufficient confidence in most clinical circumstances [9]. Schneiderman and colleagues proposed a quantitative definition of futile interventions as those that have proved useless in the last 100 cases [10], such that the physician can be confident that no more than 3% of patients would survive. However, in almost all clinical contexts, models designed to predict futility fall far short of these rigorous specifications [9]. This is a particular limitation in a medical crisis such as the current Covid-19 pandemic in which data is relatively sparse and still being collected.

Further, data used for model development may reflect informal rationing at the bedside, whereby the perceived poor prognosis in the old and the sick leads to less aggressive care—a so-called self-fulfilling prophecy [11]. Accurate prediction of futility requires prediction of outcome given maximal care, which may not be available in the data for some risk strata.

Finally, clinical practice during the Covid-19 pandemic is evolving. More frequent use of proning, minimizing paralytics, use of lung-protective volumes for ventilation, early physical therapy and other treatments (such as remdesivir) may improve prognosis over time. This learning curve can make prediction of medical futility from data even several months old, or in different settings, suspect. When medical futility is not well predicted, poor prognosis despite intervention may no longer be a reliable surrogate for the probability of benefit.

### Calibration is less important for rationing than for shared decision-making or counseling

While the need for rigorous prediction modeling methodology is increasingly recognized, when prognostic models are used for microallocation there are unique priorities for evaluation. In more familiar settings, these models are used to inform shared decision-making, with the goal of aligning therapeutic options with a patient’s personal values and preferences. In that context, ‘good calibration’ (i.e. agreement between the proportion of patients predicted to have an outcome in any strata and those that actually have that outcome) is critical for effective decision-making. Yet excellent calibration—normally the Achilles heel of prediction, since it requires consistent effects of variables not included in the model [12, 13]—is not a critical issue for microallocation. When the goal is to prioritize patients for scarce resources that would benefit a much larger group, (non-parametric) ranking (i.e. good discrimination) is key. This is because, under conditions of scarcity, the decision threshold is not determined by a patient’s (or clinician’s) point of indifference (the risk at which the utilities of alternative treatments or decisions are evenly balanced), but by capacity. That ranking is typically sufficient for microallocation should be apparent to anyone who has ever waited in a line for anything: it is unnecessary to know whether the person in front of you arrived 2 hours or 2 seconds before you.

### Fairness concerns that are not an issue when using prediction for shared decision-making emerge in the context of rationing; transparency is critical

Finally, there are a set of ‘fairness’ issues that come to the fore with rationing that are not germane in the usual shared decision-making or counseling context. Fairness concerns arise when predictions are used to adjudicate between competing interests (for example, between two patients requiring the same ventilator)—rather than to align a decision with a patient’s own values and preferences [14]. In these circumstances, predictions can be said to be ‘polar’—i.e. one pole of the probability prediction is associated with a decision that is unambiguously favorable, such that it is in the subject’s interest to get a higher (or lower) score, rather than an accurate forecast.

While models can help provide an objective basis for microallocation, they do not fully alleviate issues of group fairness. For example, age and sex are important determinants of actuarial functions, such as mortality risk and life expectancy, which may provide a basis for microallocation. If each year of life is valued similarly for all, clinical prediction will systematically prioritize treatment for the young over the old and presumably, in Covid-19 infection, for women over men. It is also not hard to see how such actuarial functions might differentially allocate resources across different racial or ethnic groups—given influential social determinants of health and other race-correlated factors—which might disadvantage historically marginalized groups. These fairness concerns may be exacerbated by machine learning approaches that are (1) highly dimensional (potentially making use of many variables that might function as proxies for race, ethnicity or sex) and (2) not open to scrutiny by clinicians, regulators or the general public. The field of algorithmic fairness has generated interesting scholarly work, but is not fully mature for application in this crisis [14].

## Conclusions

The above considerations suggest allocation methods should be explicit, data-driven, frequently updated and open to public scrutiny [15]. This latter quality argues for the use of simple and interpretable prognostic models—rather than increasingly popular ‘black box’ machine learning approaches, including many proposed in the context of Covid-19 [5].

While development of accurate clinical prediction models is important to ensure that the greatest benefit can be derived from a limited supply of life-saving resources, we should acknowledge that there will not be a perfect technical solution to this problem or a single best method of resource allocation [16, 17]. Nevertheless, when withdrawing or withholding life-saving care from patients who might potentially benefit, professional societies should aim for standardization and consensus in their guidance. It goes without saying that we hope such guidance need not be applied.

## Data Availability

Not applicable
